# Parental willingness to vaccinate adolescent daughters against human papilloma virus for cervical cancer prevention in Western Nigeria

**DOI:** 10.11604/pamj.2020.36.112.19007

**Published:** 2020-06-19

**Authors:** Haleemat Wuraola Akinleye, Oluchi Joan Kanma-Okafor, Ifeoma Peace Okafor, Kofoworola Abimbola Odeyemi

**Affiliations:** 1 Department of Community Health and Primary Care, College of Medicine, University of Lagos, Lagos, Nigeria

**Keywords:** Willingness to vaccinate, HPV vaccination, cervical cancer, adolescent girls, Surulere, Lagos, Nigeria

## Abstract

**Introduction:**

cervical cancer, which is vaccine preventable, is the commonest gynaecological cancer worldwide. This study aimed to assess parental willingness to vaccinate adolescent girls against human papillomavirus (HPV) for cervical cancer prevention.

**Methods:**

this was a descriptive cross-sectional study among 301 parents of adolescent girls who reside in Surulere Local Government Area in Lagos, Nigeria. A pretested, semi-structured interviewer-administered questionnaire was used to collect data and analysis was done using Epi-info™ version 7. The chi-square (or Fisher’s exact) test and the t-test were used to test for associations between categorical and continuous variables respectively. The level of significance was set at 0.05.

**Results:**

over half (53.5%) of the respondents had heard of cervical cancer. Of these, two thirds (62.1%) were aware that it could be prevented, 19.0% had good knowledge of cervical cancer prevention, only 4% had their daughters vaccinated though 79.2% were willing to vaccinate. The poor vaccine uptake was mostly due to lack of awareness of vaccination centres and the high cost of the vaccine. Willingness was significantly associated with level of education (p = 0.047) and knowledge of HPV vaccination (p < 0.001), however once aware, most parents were willing to get their daughters vaccinated.

**Conclusion:**

awareness about cervical cancer prevention was high though uptake was low. A high level of education and good knowledge of cervical cancer prevention were facilitators of willingness to vaccinate, though once aware parents were willing. Creating awareness and educating parents about cervical cancer prevention is essential in improving the uptake of the vaccine.

## Introduction

Cervical cancer is a malignant disease of the cervix usually occurring in the 5^th^ or 6^th^ decade of life at a mean age of 54 years, with a pre-malignant stage which usually occurs in younger women under the age of 40 [1]. Cervical cancer is the commonest gynaecological cancer in women worldwide [2]. It is one of the leading cancers among women in developing countries [3], where there were an estimated 445,000 new cases in 2012 (84% of the new cases worldwide) [4], representing approximately 13% of female cancers overall [5]. About 80% of cervical cancer cases and about 87% of deaths occur in developing countries [6]. Cervical cancer is one of the most preventable and treatable forms of cancer. Prevention and early treatment are highly cost-effective and can help to maintain the health and well-being of girls and women throughout their lives.

Human papillomavirus (HPV) is a common infection associated with genital skin to skin contact. Most women will have a transient infection only. Persistent infections are associated with integration of HPV DNA into the host cell genome, giving rise to premalignant and, less frequently, malignant change [7]. High risk HPV (hrHPV) types 16 and 18 are together responsible for around 70% of cervical cancer worldwide while the remainder of cervical cancer cases are associated with other high-risk types [8]. Controlling the risk factors: delaying first sexual intercourse until the late teens or older, HPV vaccine, limiting the number of sexual partners, avoiding sexual intercourse with people who have had many partners, not smoking, avoiding sexual intercourse with people who are visibly infected with genital warts, are major ways to prevent cervical cancer [9]. The introduction of vaccines was a point of transition in medicine. Vaccines are one of the most effective public health interventions protecting against infectious diseases. HPV vaccine prevents cancers caused by HPV infection. It is recommended for preteens (both boys and girls) aged 11 to 12 years, but can be given as early as age 9 and until age 26 for females and age 21 for males. The primary target population for HPV vaccination is girls before initiation of sexual activity. The vaccine is given in a series of either two or three doses after the recipient has been screened for HPV by a HPV DNA test, depending on age [9].

The HPV vaccination has the potential of reducing the incidence of cervical cancer worldwide by 70%; the remaining 30% of cancers can be prevented through regular cervical cancer screening [10]. The success of the vaccine would depend on levels of acceptability and uptake, which heavily rely on the willingness of parents to have their eligible daughters vaccinated [11]. Since the vaccine is targeted at adolescents who are regarded as ‘legal minors’ in Nigeria, parental consent and acceptance of the vaccines to be administered to them is an important consideration. Prior research among parents has reported predictors of vaccinations of girls to include perception of the severity of the disease, belief of daughter being at risk, higher levels of education and income, attitudes towards vaccines, previous awareness of the vaccine, and recommendations by vaccine providers [12]. Several studies that assessed parental acceptance of HPV vaccination for their daughters in Nigeria reported high levels of willingness for the vaccine [13, 14].

Barriers to HPV vaccination include high monetary cost, uncertain length of vaccine effectiveness, low perceived risk of HPV infection (since their daughters were not engaging in sex at the time), no immediate perceived need (because of the slim chance of their daughter having sex in the very near future) and the anticipated family disapproval and fear of the pain of injection. Facilitators to HPV vaccination on the other hand include having the family support needed to get one's daughter vaccinated, peer support and medical reassurance on safety and efficacy of vaccine [15]. Studies have shown that the opinion of women is strategic to HPV vaccine acceptability in various settings [12]. Some of the factors that have been found to be associated with willingness are: age, higher educational level, occupation, ethnicity, knowledge about cervical cancer, recommendation by health-care providers, religious belief and interpretation of sexual activity [16]. Various factors cause parents to choose not to vaccinate their daughters especially concerns about vaccine side effects, fear that the vaccine could be dangerous to their daughter and provider non-recommendation [17]. Studies focused on parental opinion on HPV vaccination for their children could potentially aid the successful implementation of the vaccination program because parental acceptance is a critical step in childhood vaccination. Towards that end, it is necessary to focus on the role of parents of adolescent girls in the prevention of cervical cancer. Currently, the role of mothers of adolescent daughters with respect to cervical cancer prevention primarily involves making their daughters aware of HPV and acceptance of the HPV vaccination [18]. It has been demonstrated that in developing countries mothers' awareness of HPV and cervical cancer is inadequate making it essential to educate parents to equip them to make the right decisions about their daughters' future [19]. This study aimed to determine the factors that influence parental willingness to allow HPV vaccination for their daughters so that by addressing these factors the HPV vaccination rates can be improved.

## Methods

This was a community-based cross-sectional descriptive survey conducted in July-Sept 2018, involving parents of adolescent girls in Surulere Local Government Area (LGA), Lagos, south-western Nigeria. At the last census in the year 2006, the population of Surulere was 503,975 (Lagos State Population growth rate at 3.2% per annum). With a population density of 21,864 inhabitants per square kilometre, the major occupation of the people of Surulere LGA is trading. Surulere LGA is divided into 23 administrative wards. Using the Cochrane formula, a sample size of 274 was calculated. This was increased by 10% to correct for attrition and non-response to achieve a sample size of 301. The multi-stage sampling technique was used to select the respondents for this study. In the first stage, six of the administrative wards were selected by simple random sampling using balloting. In the second stage five streets were selected from each of the six selected wards by simple random sampling also by balloting. Ten out of about 40 houses on each street were selected by systematic sampling thus, fifty houses were selected per ward. In each house, if there was more than one eligible parent present, one parent (male or female) was selected by balloting, to participate in the study.

Data were collected with the use of a pre-tested, semi-structured, interviewer-administered questionnaire developed from those used in previous studies. The questionnaire was designed to collect data on the socio-demographic characteristics of the respondents, their awareness, knowledge and attitude towards cervical cancer and cervical cancer prevention, their willingness to have their daughters receive HPV vaccination and the uptake of HPV vaccination. Data entry and analysis were done using Epi-info™ version 7 statistical software. The quantitative data was analysed as descriptive frequencies, percentages and cross tabulations. Knowledge and attitude were scored and categorised into ‘good’ or ‘poor’ and ‘positive’ or ‘negative’ respectively. The Chi-square and Fisher's exact tests were used to test for associations between categorical variables as applicable, while the t-test was used to test for associations between continuous variables. Associations were considered statistically significant if the two-tailed probability was less than 5% (0.05).

Ethical approval was obtained from the Health Research Ethics Committee (HREC) of the Lagos University Teaching Hospital (HREC Assigned NO: ADM/DCST/HREC/APP/372) prior to the commencement of the study. Permission was obtained through the Medical Officer of Health in charge of Surulere Local Government Area. A written informed consent was taken from each participant prior to their enrolment into the study. Confidentiality was ensured by keeping the data anonymous; using numbers rather than names to identify the respondents and the questionnaires. Participation in the study was purely voluntary, with no consequences for non-participation.

## Results

The age of the respondents in this study ranged from 28 to 65 years with a mean of 43.9 ± 7.3 years. More than two-thirds (69.1%) of the respondents were mothers and a third (30.9%) of them were fathers. More than half (59.1%) of the respondents were from the Yoruba ethnic group with 64.8% belonging to the Christian religion. Majority of the respondents (84.4%) were married, the mean age of their daughters being 13.5 ± 3.0 years. Over half (50.2%) of the respondents had attained tertiary education. Of the parents that participated in this study, 46.4% had one adolescent girl in their care, 39.9%, 12% and 1.7% had two, three and more than three adolescents in their care, respectively. A small proportion (4.0%) knew that their youngest adolescent daughter had experienced their sexual debut, 15.9% did not know whether or not she had, while the majority (80.1%) were certain that their daughters were virgins (Table 1). Only about half of the respondents (53.5%) were aware of such a condition as cervical cancer and of these, 62.1% were aware that it could be prevented by applying various measures including vaccination. An even smaller proportion (19.0%) among those aware of HPV vaccination had good knowledge about it (the route of administration, the frequency of vaccination, eligibility for vaccination). Nearly seven out of ten (68.0%) of those aware of the HPV vaccine had good attitude towards it.

A very small proportion (4.0%) of the parents who were aware of HPV vaccination for cervical cancer prevention actually had their daughters vaccinated, 96.0% had not vaccinated their daughters. Of those who actually did have their daughters vaccinated, 75.0% vaccinated their daughter because they were following the doctor's request. Only 1 parent (25.0%) voluntarily had their daughter vaccinated. Not being aware of how to get their daughter vaccinated (41.7%), the vaccine not being available (11.5%), the high cost of the vaccine (17.7%) and being unsure of the safety of the vaccine (11.5%) were the reasons given for not having their daughters vaccinated. Some parents (17.7%) had no reason at all for not vaccinating their daughters. Of those who had not vaccinated their daughters, a large proportion (79.2%) of the parents were willing to have their daughters vaccinated if it was provided free of charge (Table 2). Respondents level of education and level of knowledge about cervical cancer prevention showed a statistically significant association with willingness to vaccinate (p = 0.047 and p = 0.001 respectively), though the level of education showed a weak association. Willingness improved with higher levels of education and about half of those who had good knowledge were willing to vaccinate. Majority (82.7%) of those who, though aware that cervical cancer could be prevented, had poor knowledge of its prevention were still willing to vaccinate their daughters (Table 3).

**Table 1:** socio-demographic characteristics of respondents

Variable	Frequency(N=301)	%
**Age (years)**		
<30	5	1.7
30-39	111	36.9
40-49	133	44.2
50-59	44	14.6
≥60	8	2.7
**Age range**	28 - 65	
**Mean age**	43.9±7.3	
**Gender**		
Female	208	69.1
Male	93	30.9
**Ethnic group**		
Yoruba	178	59.1
Hausa	19	6.3
Igbo	76	25.2
Others	28	9.3
**Religion**		
Christianity	195	64.8
Islam	105	34.9
Traditional	1	0.3
**Marital Status**		
Single	27	9.0
Married	254	84.4
Widowed	7	2.3
Divorced	8	2.7
Separated	5	1.7
**Level of education**		
No formal education	18	6.0
Primary	28	9.3
Secondary	104	34.6
Tertiary	151	50.2
**Occupation**		
Unemployed	5	1.7
Unskilled	142	47.2
Skilled	35	11.6
Professionals	119	39.5
**Number of adolescent girls in your care**		
1	140	46.4
2	120	39.9
3	36	12.0
>3	5	1.7
**Sexual debut of youngest adolescent**		
Yes	12	4.0
No	241	80.1
I don't know	48	15.9

**Table 2:** respondents awareness, knowledge, perception and uptake of cervical cancer prevention

Variable	Frequency	%
**Awareness of cervical cancer(n=301)**		
Yes	161	53.5
No	140	46.5
**Awareness of cervical cancer prevention(n=161)**		
Yes	100	62.1
No	61	37.9
**Overall knowledge of cervical cancer prevention(n=100)**		
Good	19	19.0
Poor	81	81.0
**Overall attitude towards cervical cancer prevention (n=100)**		
Positive	68	68.0
Negative	32	32.0
**Uptake of HPV vaccine**		
**A) Daughter vaccinated (n=100)**		
Yes	4	4.0
No	96	96.0
**B) Reason for receiving vaccine (n=4)**		
Doctor's request	3	75.0
Voluntary	1	25.0
**C) Reason for not receiving vaccine (n=96)**		
Does no know any vaccination centres	40	41.7
Vaccine not available	11	11.5
High cost of vaccine	17	17.7
Worried about vaccine safety	11	11.5
No reason	17	17.7
**Willingness to get daughter vaccinated (n=96)**		
Yes	76	79.2
No	20	20.8

**Table 3:** factors associated with willingness to vaccinate daughter (if vaccine was free)

Variable	Willingness to vaccinate daughter		X^2^	P-value
	**Yes n = 76 Freq (%)**	**No n = 20 Freq (%)**		
**Age (years)**				
**<** 39	21(87.5)	3(12.5)		0.320*
40-49	39(79.6)	10(20.4)		
≥ 50	16(69.6)	7(30.4)		
**Gender**				
Male	22(84.6)	4(15.4)		0.575*
Female	54(77.1)	16(22.9)		
**Marital Status**				
Married	61(80.3)	15(19.7)	0.27	0.612
Not married	15(75.0)	5(25.0)		
**Ethnic group**				
Yoruba	45(80.4)	11(19.6)	0.12	0.734
Non-Yoruba	31(77.5)	9(22.5)		
**Religion**				
Christianity	57(76,0)	18(24.0)		0.133*
Others	19(90.5)	2(9.5)		
**Level of education**				
Primary/Secondary	9(60.0)	6(40.0)	3.96	**0.047**
Tertiary	67(82.7)	14(17.3)		
**Number of adolescent girls in your care**				
1	37(78.7)	10(21.3)	0.01	0.917
> 1	39(79.6)	10(20.4)		
**If youngest adolescent daughter is sexually active**				
Yes	1(50.0)	1(50.0))		0.189*
No	61(77.2)	18(22.8)		
I don't know	14(93.3)	1(6.7)		
**Knowledge of cervical cancer prevention**				
Good	9(47.4)	10(52.6)	14.52	**< 0.001**
Poor	67(87.0)	10(13.0)		
**Attitude toward cervical cancer prevention**				
Positive	51(76.1)	16(23.9)		0.412*
Negative	25(86.2)	4(13.8)		

* Fisher's exact p-value

## Discussion

Cervical cancer is an important disease that requires attention even at the time of adolescence, keeping prevention in view. Not many parents know about the vaccines nor are getting their daughters vaccinated. The analysis of the demographic data showed a higher percentage of females among the parents, reflecting the fact that mothers are usually the parent available to care for children even at adolescence. This study demonstrated that an average proportion of the parents were aware of cervical cancer, this can be compared to the findings of a study conducted in North West Ethiopia where 69.3% had heard about cervical cancer [20]. A study conducted among mothers in Somolu, an urban area in Lagos, found that 79.6% had heard about cervical cancer [21], however in another study in urban Lagos a lower proportion (37.2%) of respondents had heard about cervical cancer [22]. In the current study, the major source of information was the media (TV/Radio/Newspaper/Social media), this is similar to a study in an urban neighbourhood in Lagos where the commonest source of information about cervical cancer was the television (39.0%) [22]. More than half (62.1%) of the respondents in this study were aware that cervical cancer could be prevented by HPV vaccination, which is much higher than was found in Ilorin, another South-western State in Nigeria, where 35.1% were aware of HPV vaccine for the prevention of cervical cancer [23]. The cosmopolitan nature of Lagos might have accounted for this difference as more parents likely had more information on cervical cancer and its prevention.

In this study, almost half of the respondents were aware of HPV infection. This is in contrast to a study in Bamako, Mali where awareness about HPV was much lower, with only 8.6% knowing about HPV as an STI [24]. This could be as a result of the spatial differential as the Bamako study was conducted in a peri-urban area. However, in a similar study conducted in Turkey among female adolescent and young women the proportion was similar (41.6%) [25]. The current study showed an above average level of awareness of cervical cancer prevention with HPV vaccine, higher than in a previous study carried out in Indonesia with awareness of HPV vaccines of 44% among parents [26] These studies have shown that there is generally a low level of awareness of HPV vaccine in most parts of the developing world. In this study, majority had poor knowledge of cervical cancer prevention including HPV vaccination, which is similar to a study in an urban area in Lagos where 90% had poor knowledge [21], and another study in Chicago, USA where 73% had poor knowledge [27]. However, in another study in urban Lagos, 81.5% knew that HPV vaccination prevents cervical cancer [22]. This difference may be due to the increased access to information as provided by the more formal workplaces of the respondents in the Somolu study. Majority (68.0%) of the respondents had positive attitude towards cervical cancer prevention, which is similar to a study conducted in Zaria, North east Nigeria where 80.4% of the respondents had a positive attitude towards cervical cancer prevention [28]. The generally positive attitude towards cervical cancer prevention may be due to the general opinion held about cancers as being deadly.

In this study, only 1.3% of the respondents had adolescent daughters who had received HPV vaccine despite the fact that they were aware of the vaccine. However, 80.9% of the parents were willing to have their daughters vaccinated. This is similar to a study carried out in Lagos where only 2.2% of the respondents had HPV vaccine given to their female teenage children and this was despite the acceptance rate of HPV vaccination being 76.2% among the respondents [29]. In another study in Nigeria, most of the respondents (65.7%) supported vaccination of adolescent girls [24]. Majority of the parents (41.7%) who had never had any of their daughters vaccinated attributed this to lack of awareness of the vaccine and vaccination centres. This is comparable to a study in Mushin, Lagos where the vast majority of the women (54.1%) who had never had any of their children vaccinated attributed this to lack of awareness of the existence of the vaccine and its benefits [30].

The high level of willingness shown by the respondents in this study (80.9%) to accept HPV vaccination for their daughters is consistent with the reports from previous studies in Poland among parents where a major proportion of the total respondents (85.1%) expressed their willingness to vaccinate their daughters against HPV infection [31]. This is also similar to another study in UAE where 76.6% of the respondents were willing to give their daughters HPV vaccine [32] and another study in Enugu, Nigeria where 91% of the respondents were willing to recommend it to their adolescent daughters [14]. Reasons for non-vaccination against HPV were lack of awareness, lack of availability, worry about its safety, and the high cost, while some respondents gave no specific reason for non-vaccination. In a study carried out among women attending a gynecological outpatient clinic, similar reasons were found to influence the willingness to vaccinate against HPV [30].

In another similar study carried out in Bangladesh, similar but additional factors influencing willingness to vaccinate against HPV were found which included lack of awareness (78.4%), doctors not recommending the vaccine (13.6%), worries about safety (6.8%), considering the recipient as being too old for vaccination (5.7%), not needing a vaccine (3.4%), not being sexually active (3.4%) and the vaccine being too expensive (2.3%) [33]. In this study, over 80% of those with tertiary education showed willingness and about half of the parents with good knowledge of cervical cancer prevention were willing to vaccinate their daughters. A study in Poland supports this finding by reporting that willingness was influenced by having previous information about HPV (OR 2.02; 95% CI: 1.17-3.51) [31]. Also, majority of the parents despite not having adequate knowledge about cervical cancer prevention were willing to have their daughters vaccinated. This is probably due to the fact that given the seriousness of the disease and people's fear of cancer, they did not need to have good knowledge to seek to protect their daughters from cervical cancer.

## Conclusion

The parents had good attitudes towards cervical cancer prevention, despite poor knowledge. The uptake of HPV vaccination was very poor mostly due to not knowing the vaccination centres, the high cost of the vaccine and the fear of side effects. Willingness was however high especially if vaccines were to be made available free of charge. Though having good knowledge was important, parents once aware of cervical cancer prevention were willing to vaccinate their daughters, hence improving public awareness about the vaccines and the vaccination centres, and reducing the cost of the HPV vaccine is key to improving vaccine uptake. Further studies, qualitative in nature, are required to identify targeted worries and expectations of potential users and parents/caregivers.

### What is known about this topic

- Cervical cancer, the commonest gynaecological cancer in women worldwide and the second most common cancer in women living in less developed regions, is preventable;
- Awareness and access to HPV vaccination is poor in Nigeria.

### What this study adds

- There is a low level of uptake of adolescent HPV vaccine, even though most parents are willing to have their daughters vaccinated;
- Awareness even without having good knowledge is sufficient in improving parental willingness to vaccinate their daughters;
- Taking steps to ensure public awareness about cervical cancer prevention and reducing the cost of the vaccine could improve uptake of HPV vaccination.
